# Nano-engineering safer-by-design nanoparticle based moth-eye mimetic bactericidal and cytocompatible polymer surfaces†

**DOI:** 10.1039/c8ra03403f

**Published:** 2018-06-20

**Authors:** Felipe Viela, Iván Navarro-Baena, Alejandra Jacobo-Martín, Jaime J. Hernández, Marta Boyano-Escalera, Manuel R. Osorio, Isabel Rodríguez

**Affiliations:** Madrid Institute for Advanced Studies in Nanoscience (IMDEA Nanoscience) C/Faraday 9, Ciudad Universitaria de Cantoblanco Madrid 28049 Spain i.rodriguez@imdea.org

## Abstract

Nanotechnology provides a new design paradigm for alternative antibacterial strategies in the fight against drug-resistant bacteria. In this paper, the enhanced bactericidal action of moth-eye nanocomposite surfaces with a collaborative nanoparticle functional and topography structural mode of action is reported. The moth-eye nanocomposite surfaces are fabricated in combined processing steps of nanoparticle coating and surface nanoimprinting enabling the production of safer-by-design nanoparticle based antibacterial materials whereby the nanoparticle load is minimized whilst bactericidal efficiency is improved. The broad antibacterial activity of the nanocomposite moth-eye topographies is demonstrated against Gram-positive *Staphylococcus aureus* and Gram-negative *Escherichia coli* and *Pseudomonas aeruginosa* as model bacteria. The antibacterial performance of the moth-eye nanocomposite topographies is notably improved over that of the neat moth-eye surfaces with bacteria inhibition efficiencies up to 90%. Concurrently, the moth-eye nanocomposite topographies show a non-cytotoxic behaviour allowing for the normal attachment and proliferation of human keratinocytes.

## Abstract 2

Moth-eye nanocomposite surfaces are fabricated in combined processing steps of nanoparticle coating and surface nanoimprinting enabling the production of safer-by-design antibacterial nanoparticle-based materials.

## Graphical abstract

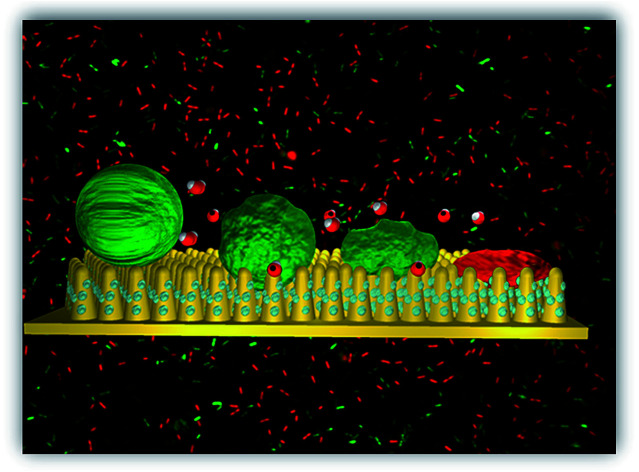

## 1. Introduction

Bacteria colonization of surfaces is not only a source of serious concern in the healthcare and biomedical fields but also impacts adversely industries such as food packaging, water purification, furnishing, construction or shipping.

Despite the multiple approaches to combat infections, bacterial colonization still remains a serious health and economic problem in our society. On the other hand, the extensive use of antibiotics and biocides has led to the development of multi-drug resistant bacteria or superbugs.^1^ The rise of superbugs today has emerged as one of the world's greatest health threats for the inability to control these infections using the existing drugs with the associated increase in morbidity and mortality and the risk of spread into epidemics.

In the face of the superbug threat, new antibacterial strategies from different fronts are being urgently sought to stop bacterial infection and the rising of resistance.^2,3^ The most effective and practical approach to prevent the development of resistance is clearly the prevention of infection by methods that do not generate resistance. Methods that are less likely to induce resistance are principally those with a physical mode of action which do not target bacteria biochemical pathways.

Among the physical anti-infective methods, biomimetic micro and nano topographies have emerged as a new approach to control bacteria attachment and proliferation onto a surface. The nanotopography of the cicada wing was the first demonstration of the bactericidal capability of these surfaces due to mechanical effects.^4–6^ Subsequently, other biomimetic topographies have been reported to be bactericidal, including the dragonfly,^7,8^ damselfly^9^ and planthopper wings,^10^ gecko skin^11,12^ and the moth-eye topography.^13^ Different materials have been employed to fabricate these surfaces such polymers,^13,14^ black silicon,^15^ titanium, titanium oxide^16^ and black titanium.^17^ The biomimetic bactericidal approaches described so far have been summarized in recent extensive reviews.^18–20^ The reported bactericidal efficacy of biomimetic soft surfaces typically oscillates about the 50% of the initial bacterial load attached to the surface.^13,14,19–21^

Another interesting non-target specific anti-infective strategy is bacteria inhibition through oxidative damage induced by reactive oxygen species (ROS). Metal oxide nanoparticles (NPs) such as TiO_2_, ZnO and CuO being wide band-gap semiconductors, have proven to be very effective ROS generators. ROS comprise of highly reactive radicals (superoxide anion (˙O_2_^−^), hydroxyl radicals (˙OH), hydrogen peroxide (H_2_O_2_) and organic hydroperoxides) which are able to cause oxidative damage to practically all biomolecules including protein and lipid components in the cell membrane.^22^ As such, metal oxide NPs have emerged as a promising non-target specific broad spectrum anti-infective strategy and a less toxic alternative to metal biocides such as the commonly employed silver NPs.^23^

Metal oxide NPs have the advantages of being low cost, stable and of low toxicity. As such, TiO_2_ and ZnO are currently used extensively in personal care products such as sunscreens and cosmetics. Even though, these NPs do not appear to cross the skin,^24^ there are still concerns on their safety for humans because the mechanisms of action are still not fully known.^25^ Moreover, several studies have reported their eco-toxicity due to the destruction of other organisms including earth autochthonous microflora (bacteria, fungi, algae) and non-target organisms such as aquatic species.^26,27^ With the widespread use and increasing production of metal oxide NPs, the potential for the uncontrolled release of large amounts of NPs to the environment undoubtedly has increased which is certainly a reason for concern.

Accordingly, until the toxicity mechanisms of the metal oxide NP are completely unravelled, there is a need to design products with contained toxicity and with reduced environmental impact; products that enable the safe disposal of NP or their recovery for recycle.^28^

In this context, nanoparticle-polymer composite materials are emerging as a well-suited route to utilize NPs as biocides in wide fields of application.^29^ Nanocomposites containing the NPs embedded within the matrix not only broaden the physical properties and functionality of the polymers but in addition, with NPs embedded, the toxicity of the free NPs is considerably reduced and the uncontrolled release to the environment minimized.^30^

The antibacterial action of several nanocomposites has been reported before.^31^ On these, metal oxide particles such TiO_2_ and ZnO and to a lesser extent CuO have gained a great deal of interest as fillers due to lower toxicological concerns. Synthetic polymers such polyester or polyacrylate matrices filled with TiO_2_ and ZnO have shown to be effective antibacterial nanocomposites against Gram positive and Gram negative bacteria.^32,33^ Biological polymers such as chitosan or cellulose have been combined with CuO and TiO_2_ NPs to effectively reduce bacterial proliferation.^34–36^ These results show that albeit embedded on a polymer matrix, NP_S_ can retain their bactericidal action.

The cytotoxicity of ZnO and TiO_2_ nanocomposites has been also investigated in few studies.^37^ Schwartz and co-workers found ZnO polymer composites non-cytotoxic towards a mammalian cell line at bactericidal loadings.^30^ Wu and co-workers, found negligible the cytotoxicity of nanocomposites containing poly(lactic-*co*-glycolic) and TiO_2_ NP_S_ with concentrations up to 10%.^32^

Here, we report a practical processing method based on nanoimprinting replication to fabricate moth-eye mimetic anti-bacterial nanocomposite surfaces with enhanced bactericidal efficacy. The new processing method achieves efficient NPs – surface matrix dispersion and topography imprinting in a single step. Accordingly, the method permits decreasing the NPs load as it is restricted and contained within the effective patterned surface. The NPs in combination with the topography gave rise to a strong bactericidal action against Gram-positive and Gram-negative model bacteria when irradiated with UV light in the case of TiO_2_ NPs and in the dark for ZnO achieving bacteria inhibition rates from 60% to 90%. The cytocompatibility of the PMMA–ZnO bactericidal moth-eye imprinted nanocomposite towards human keratinocytes was also studied and demonstrated.

The results of this work reveal moth-eye bactericidal nanocomposites as an emerging, safer-by-design efficient antibacterial material with potentially high cytocompatibility.

## 2. Experimental section

### 2.1 Synthesis of TiO_2_ NPs

TiO_2_ NPs were synthesized by the hydrothermal method described before by Burnside *et al.*^38^ For this, 20 ml of titanium isopropoxide(iv) (Acros Organics) was added to 36 ml of deionized water and the mixture was stirred for one hour. The resultant product was filtered and washed three times using deionized water. After filtration, the solid obtained was placed into a Teflon lined hydrothermal synthesis reactor and mixed with 3.9 ml of 0.6 M tetramethylammonium hydroxide (Sigma Aldrich). The reactants were placed in an oven at 120 °C for 14 hours. The resultant colloid was centrifuged two times at 10 000 rpm for 10 min to remove aggregates. The obtained aqueous dispersion contained a NP concentration of 24% (wt/v) with a diameter of 24 nm as measured by dynamic light scattering (DLS) (Malvern Zetasizer) (See Fig. S1†). For the fabrication of nanocomposite surfaces, a dispersion of the TiO_2_ NPs in methanol (0.5 wt/v%) was prepared. The X-ray diffraction patterns of synthesized TiO_2_ and ZnO nanoparticles utilized in this work can be seen in Fig. S2.† ZnO diffraction peaks indicate a typical single-phase hexagonal wurtzite lattice. TiO_2_ lattice parameters match well with those of anatase phase.

### 2.2 Modification of ZnO NPs

ZnO NPs with an average diameter of 20 nm (Nanoamor) were silanized to improve their dispersion. For this, 1 g of ZnO NPs was dispersed in 100 ml of deionized water by ultrasonication for 10 min. Then 1 ml of the silane agent, 3-aminopropyltriethoxysilane (APTMS, Sigma-Aldrich) was added to the dispersion and the mixture was stirred for 24 hours at 95 °C. The dispersed NPs were then separated from the solvent by centrifugation at 10 000 rpm for 10 min and subsequently washed with methanol to remove the excess of silane. After, the modified particles were dried in vacuum at 100 °C for 24 h. The modified ZnO NPs were dispersed in methanol by ultrasonication at a concentration of 0.5 wt/v%.

### 2.3 Fabrication of PMMA moth-eye patterned nanocomposites

The patterned nanocomposites were fabricated on poly(methyl methacrylate) (PMMA). Initially, PMMA thin films were produced on glass cover slips of 18 mm in diameter. The glass cover slips surfaces were first activated with oxygen plasma (Tepla 600) at 300 W for 5 min to improve the adhesion and then, a solution of PMMA (*M*_w_ 120.000, Sigma-Aldrich) on toluene (7.5 wt/v%) was spin-coated at 1000 rpm for 1 min and the resultant film annealed at 100 °C. Subsequently, the films were activated with oxygen plasma (Tepla 600) at 50 W for 1 min and the prepared (0.5 wt/v%) TiO_2_ or ZnO NP dispersions spin coated. On the PMMA-NP prepared films, the moth-eye nanocomposite structures were nanoimprinted at 170 °C and 45 bars of pressure for 5 min using an Eitre 3 Nanoimprint lithography system (Obducat Technologies AB) using a PDMS working mould. As control substrates, smooth nanocomposites were prepared following the same conditions but pressed using instead a flat slab of PDMS. The PDMS working mould was obtained by replication of a master nickel mould (HT-AR-02, Temicon) as reported before.^13^ The nanocomposite substrates were imaged by scanning electron microscopy (SEM) using an Auriga FIB-SEM system (Zeiss) and by atomic force microscopy (Multimode 8 AFM system, Bruker).

### 2.4 Determination of free NP released from the nanocomposites

To determine the possible release of NPs into the media, the nanocomposite imprinted substrates were immersed on 2 ml of phosphate-buffered saline (1× PBS) at 37° and stirred for 7 h. On the solutions, DLS and absorbance measurements were performed.

### 2.5 Determination of free Zn and Ti ions released from the nanocomposites

The concentration of free ions in the media was determined by inductively coupled plasma-mass spectroscopy (ICP-MS) in phosphate buffer (PB) and on Luria–Bertani (L–B) media by incubating the nanocomposite imprinted substrates in these solutions at 37 °C during 7 hours.

The PB buffer was prepared at pH 7.6 with a concentration of 0.1 M by mixing 100 ml of a 27.6 g L^−1^ solution of NaH_2_PO_4_ (Sigma-Aldrich) and 400 ml of a 28.4 g L^−1^ solution of Na_2_HPO_4_ (Sigma-Aldrich). Prior to incubation, the PB buffer solution was diluted 1000 fold and the LB media 500 fold to reduce the sodium content as it interferes with the detection. After the incubation time, solutions were collected for elemental analysis by ICP-MS (NexION 300XX, Perkin-Elmer).

### 2.6 Detection of ROS production

To verify the production of hydroxyl radicals from the ZnO composite surfaces in the dark, fluorescence spectroscopy of therephthalic acid (TA) (Sigma-Aldrich) as trap agent was employed. In a typical procedure, the nanocomposite imprinted slips were immersed in 2 ml of 2 mM TA solution in 1× PBS and stirred in the dark. At regular intervals, the fluorescence emission of the solution was read at excitation wavelength of 312 nm.

The detection of H_2_O_2_ was carried using Ampliflu Red (AR) (Sigma-Aldrich (98%)) as fluorescence probe. The fluorescent assay relies on the horseradish peroxidise-catalysed reaction of H_2_O_2_ and Ampliflu Red with a 1 : 1 stoichiometry to form the coloured, fluorescent resorufin. Hydrogen peroxide (30 wt%) and horseradish peroxidase (HRP, type VI), were purchased from Sigma-Aldrich. The fluorescence product was monitored at the excitation wavelength of 550 nm and emission wavelength of 580 nm. The H_2_O_2_ working solutions to obtain a calibration curve were prepared by serial dilutions of a 0.1 M H_2_O_2_ stock solution with MilliQ water. The reactions were carried out in 20 mM HEPES buffer with pH adjusted to 8.1.

For the detection of H_2_O_2_, the imprinted nanocomposites substrates were introduced in a cuvette with 3 ml of the reaction solution containing 2 μM of AR and 1.24 U mL^−1^ of HRP in 20 mM HEPES. The reaction solution was stored in the dark at room temperature and the fluorescence was determined at increasing time intervals.

### 2.7 Bacteria culture and live/dead viability assays

*Escherichia coli* (CECT 516), *Pseudomonas aeruginosa* (CECT 4628) from the Spanish Type Culture Collection (CECT) (Valencia University) and *Staphylococcus aureus* (RN 4220) were cultured on the prepared substrates to assess the antibacterial properties of the topography following protocols reported before.^13^ After the incubation period, the TiO_2_ surface nanocomposites were exposed to UV light with a maximum intensity at 356 nm, *i.e.* in the UV-A region non-hazardous to bacteria with a lamp (UVASPOT 400/T, Honle) providing 80 mW cm^−2^ of intensity for 2 min. Then, the substrates were gently rinsed using 1× PBS (Fisher Scientific) and stained using 0.13 μl of the staining solution (Live/Dead® Baclight™ Viability Kit (Molecular Probes)) for 1 ml of Tris–HCl for 15 min in the dark at room temperature. Lastly, the substrates were rinsed with 1× PBS and mounted with BacLight mounting oil. Live and dead bacteria were counted from fluorescent images using ImageJ image analysis software (NIH). Four independent trials were run with three replicates of each substrate.

### 2.8 Bacteria morphology imaging

Scanning electron microscopy (SEM) images were taken to visualize the attachment of bacteria onto the moth-eye nanocomposites surfaces. Prior imaging, all substrates were fixed following protocols reported before.^13^ The substrates were sputter-coated with a thin layer of gold and imaged on an Auriga FIB-SEM system (Zeiss).

### 2.9 Cellular toxicity of ZnO-nanocomposites

Cell proliferation assays and morphology analysis were performed using human keratinocytes (HaCaT cells) following protocols reported before.^13^

## 3. Results and discussion

### 3.1 Moth-eye mimetic nanocomposite surfaces fabrication and characterization

For the fabrication of the antibacterial moth-eye patterned nanocomposites, a new practical process was implemented that allowed creating nanocomposite surfaces and moth-eye patterns in a single processing step. PMMA was employed as matrix because it is a polymer widely used in orthopaedic applications and in ocular implants.^39^ It is also a commodity plastic used in a wide range of products. PMMA as thermoplastic, it is readily processable by thermal nanoimprinting. The fabrication of the moth-eye patterned nanocomposites starts by layering a PMMA solution onto glass cover slips by spin coating. Subsequently, a methanol dispersion of the NPs is spin-coated onto the PMMA film. In the next step, the films are imprinted in a thermal process using a moth-eye patterned PDMS replica mould. After cooling, the films are de-moulded obtaining the polymer moth-eye structures with embedded NPs. (a schematic of the fabrication process is shown in Fig. S3†).

**Fig. 1:**
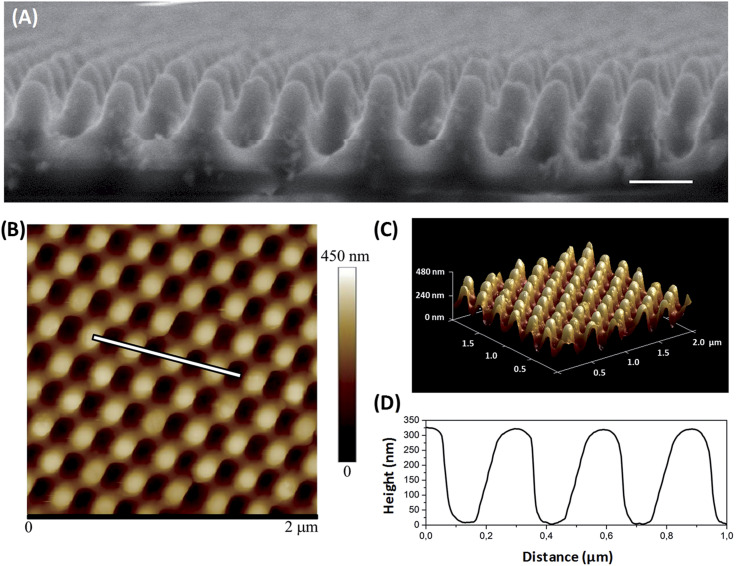
Moth-eye mimetic nanopatterned TiO_2_ nanocomposite images and geometrical characterization by (A) SEM, (scale bar 200 nm) and (B) by AFM. (C) Corresponding 3D AFM image reconstruction and (D) AFM cross sectional profile.

The quality of the replication was assessed by SEM and AFM imaging. As displayed in Fig. 1(A) and (B), the SEM and AFM images of the nanoimprinted TiO_2_ nanocomposite substrates revealed a topography of well-defined nanocones disposed on hexagonal arrangement with the NPs distributed and embedded within the nanocone surface. From the AFM images, the height of the topography was determined to have a mean value of 310 nm and a feature width on the cap of 80–100 nm and a pitch of 250 nm (Fig. 1(C)). Due to the presence of the NPs during the imprint process, the height of the nanocones was reduced slightly from that of the mould (350 nm). Nonetheless, the nanocone features were well formed and maintained a high aspect ratio of 3.8. The topography imprinted on the ZnO nanocomposite surfaces showed a mean height of 275 nm (See Fig. S4†).

### 3.2 Antibacterial effect of moth-eye mimetic nanocomposites

To determine the bactericidal activity of the TiO_2_ and ZnO composite moth-eye surfaces, the viability of *E. coli*, *P. aeruginosa* and *S. aureus* was assessed as model of Gram negative and Gram-positive bacteria. After the defined incubation periods on the different substrates, the live and dead bacteria attached on to the surfaces were fluorescently stained and counted on fluorescent microscopy images.

**Fig. 2:**
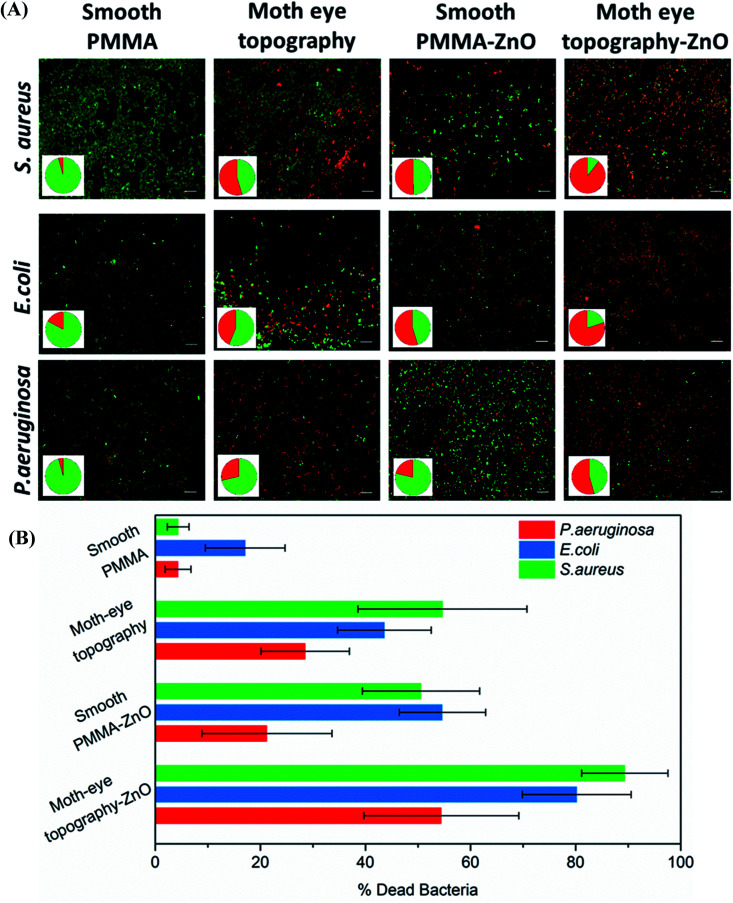
Bactericidal efficiency of moth-eye mimetic ZnO nanocomposite topography. (A) Comparative fluorescence imaging of live and dead *S. aureus*, *E. coli* and *P. aeruginosa* bacteria incubated on smooth PMMA and PMMA–ZnO surface nanocomposite and on PMMA and PMMA–ZnO nanocomposite moth-eye imprinted topographies. Scale bar 5 μm. Pie charts show the percentages of live and dead bacteria (green and red, respectively). (B) Bactericidal efficacy plots. Error bars represent standard deviation.

Fig. 2 shows representative fluorescence images of the different live–dead bacteria populations observed on the PMMA–ZnO moth-eye imprinted and smooth nanocomposites and smooth and imprinted PMMA control substrates. The results reveal a bactericidal efficacy of around 50% for *S. aureus* and *E. coli* and 30% for *P. aeruginosa* of the smooth PMMA–ZnO nanocomposite surfaces and similar for the case of the moth-eye mimetic topography. However, when bacteria were cultured onto the ZnO-nanocomposites moth-eye imprinted substrates, the population of dead bacteria increased up to 90% for *S. aureus,* 82% for *E. coli* and 55% for *P. aeruginosa*, underlying the collaborative bactericidal effect of the topography with the ZnO NPs.

Since TiO_2_ has a large band gap of 3.0–3.3 eV, only under the action of UV irradiation, photo-activation and production of free radical species is possible.^31^ Nonetheless, to verify this point, the bactericidal effect of the smooth PMMA–TiO_2_ composite in absence of UV light comparatively to that of the neat PMMA polymer as negative control was initially examined and no evidence of bactericidal action was found (See Fig S5†).

A clear bactericidal activity of the PMMA–TiO_2_ composite surfaces was observed when the substrates were irradiated with UV light after the incubation period. The time of exposure was set at 2 min when an evident bactericidal action was observed.

**Fig. 3:**
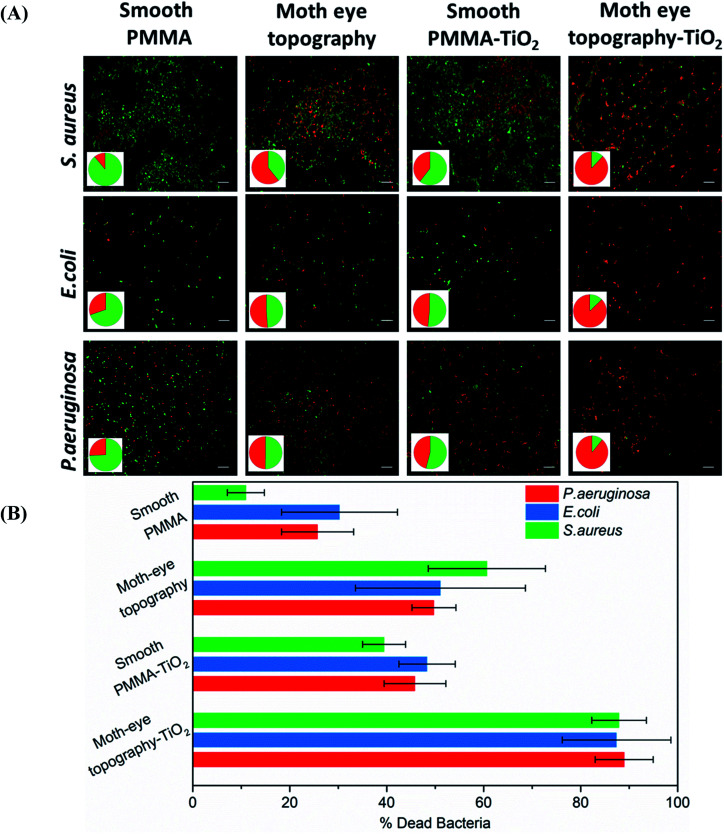
Bactericidal efficiency of moth-eye mimetic TiO_2_ nanocomposite topography. (A) Comparative fluorescence imaging of live and dead *S. aureus*, *E. coli* and *P. aeruginosa* bacteria incubated on smooth PMMA and PMMA–TiO_2_ surface nanocomposite and on PMMA and PMMA–TiO_2_ surface nanocomposite moth-eye imprinted topographies. Scale bar 5 μm. Pie charts show the percentages of live and dead bacteria (green and red, respectively). (B) Bactericidal efficacy plots. Error bars represent standard deviation.

Fig. 3 shows representative microscopy images of the fluorescently labelled live and dead bacteria populations observed on the PMMA–TiO_2_ moth-eye imprinted and smooth nanocomposites and on the smooth and imprinted PMMA neat substrates employed as controls. The results reveal a reduction of the bacterial load about 50–60% for both of the moth-eye PMMA topography and the smooth PMMA–TiO_2_ nanocomposite upon UV illumination. On the other hand, this percentage increased up to 90% in the moth-eye PMMA–TiO_2_ patterned nanocomposite substrates for the three bacteria tested.

### 3.3 Bacteria-surface interaction: bactericidal mechanism

**Fig. 4:**
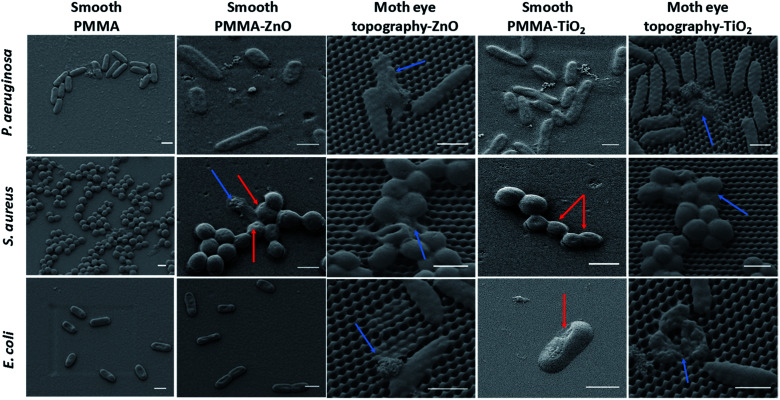
Comparative SEM imaging of the bacteria-topography interaction of *S. aureus*, *E. coli*, and *P. aeruginosa* attached onto smooth PMMA nanocomposites and moth-eye mimetic nanopatterned nanocomposites. Scale bar 1 μm.

The morphological observation by SEM imaging of the bacteria attached onto the surfaces of smooth and moth-eye patterned nanocomposites in Fig. 4 reveals distinct morphological changes on the adhered bacteria. On the nanocomposite flat surfaces, the oxidative damage is clearly visible where it can be appreciated some bacteria displaying a rough surface and cavities on their membrane (red arrows). On the nanopatterned nanocomposites, the dead bacteria show predominantly a significant loss of morphology, exhibiting the broad and crushed appearance found before on bactericidal natural or biomimetic topographies.^6,13^ But in addition, in this case, some bacteria exhibit a complete released of the cytoplasm content and substantial membrane degradation (blue arrows). Thus, from the fluorescence and SEM images, a collaborative action of moth-eye mimetic topography and NPs can be recognized.

Different mechanisms have been put forward in the literature to account for the bactericidal effect of metal oxide NPs, these include direct contact of NPs with the bacteria wall resulting in destruction of bacteria cell integrity or endocytosis followed by disruption of the cellular activities,^37^ liberation of positive ions and ROS formation.^40,41^ Nonetheless, the mechanisms of toxicity of metal oxide NPs still remain under debate.^40^

Therefore, control experiments were carried out in order to get further insight into the dominant biocidal mechanism that applies in the case of the moth-eye patterned nanocomposites.

To determine a possible bactericidal action of NPs freed from the imprinted nanocomposite surfaces, release experiments of NPs to the aqueous media were performed. At the detection limit of the DLS tool employed, the release of NPs was not detected from the ZnO or the TiO_2_ composite imprinted surfaces and as such, a bactericidal action from free NPs can be considered insignificant in this case.

Some authors have ascribed the bactericidal action of ZnO to the release of free Zn^2+^ ions due to the dissolution of ZnO nanoparticles.^42^ Free Zn^2+^ ions have been postulated to be toxic for bacteria because the interaction between Zn^2+^ ions and bacteria can destroy the charge balance of the outer membrane cell wall, leading to membrane deformation and ultimately bacteriolysis.^22^

To ascertain this possible effect on bacteria, the concentration of ions dissolved from the nanocomposite imprinted surfaces was determined by ICP-MS (See Table S6†). The concentration of Zn^2+^ ions released from the ZnO imprinted surfaces in PB buffer were less than 0.5 mg L^−1^ and 0.04 mg L^−1^ on LB medium. It is noted that the concentration of Ti^4+^ ions released from TiO_2_ imprinted nanocomposites was insignificant. The release of Zn^2+^ possibly comes from partial dissolution of the ZnO NPs that are not fully covered by the polymer matrix in the composite. As noted by previous authors, the solubility of ZnO increased in PB buffer since anionic components promote its dissolution.^22^ This concentration is much lower than the bacteria inhibitory concentration determined in previous works at concentrations above 10–20 mg L^−1^.^43^ However, it cannot be ruled out that the Zn^2+^ could play a part on the antimicrobial activity observed if local higher concentration gradients are generated at the surface as ions are released.

Next, we investigated the bactericidal action of TiO_2_ and ZnO NPs due to photoinduced catalytic reactions and resulting formation of ROS. Exogenous ROS at high concentration can produce lipid peroxidation and protein damage causing destruction of the bacteria membrane leading to bacterial death.^41,44^

The bactericidal activity of TiO_2_ nanocomposites through the generation of ROS under UV light exposure has been documented before.^31,45^ Similarly in this work, with TiO_2_ NPs present on the topography and 2 min UV exposure, an intensive bactericidal action was obtained.

Hence, we particularly focused on investigating the bactericidal effect of ZnO due to ROS-induced oxidative stress arising from the production of ROS during the dark incubation conditions implemented in our experiments. The origin of ROS in the dark has been attributed to the crystal lattice point defects on the surface of ZnO crystal.^46^ Particularly surface oxygen vacancies (SOV) are common native point defects responsible for trapping electron carriers and as such, these defects behave as electron deep donors.^47^ (The photoluminescence spectra of the ZnO NPs employed exhibiting the presence of defects is shown in Fig. S7†). The reduction of oxygen by electrons released from the SOV has been proposed as the starting point in the production of ROS under dark conditions in water media.^48^ Prasanna *et al.*,^49^ described the mechanism of ROS generation in the dark and proposed that the oxygen present in the media captures electrons originated from the SOV to form superoxide anion radicals (˙O_2_^−^). A superoxide radical in water solvates to form a hydroperoxyl radical (˙HO_2_), and the latter can recombine to form H_2_O_2_. H_2_O_2_ can react with a superoxide anion radical to form a hydroxyl radical (˙OH) and a hydroxyl ion (OH^−^). Since holes are not produced, the generation of singlet oxygen is not possible in the dark, as they indicated. The authors have also found direct correlation between the number of SOVs present on the surface as measured by X-ray photoelectron spectroscopy with the concentration of ROS formed.^50^

The production of hydrogen peroxide (H_2_O_2_) and hydroxyl radicals (˙OH) by the ZnO moth-eye patterned nanocomposites in the dark was investigated through fluorescence spectroscopy. The hydroxyl radical formation was detected using terephthalic acid (TA) as trapping agent. TA reacts with ˙OH and forms 2-hydroxyl terephthalic acid (HTA) whose fluorescence emission correlates to the hydroxyl radical concentration. Fig. S8(A and B)† show the emission spectra of the TA and that of the HTA formed upon incubation of the substrates in the dark. It can be noted that the fluorescence emission increased weakly but sufficient for detection over a period of 7 h. Plotting the HTA generation kinetics (see Fig S7(C)),† it can be seen that the emission intensity increased linearly, indicating the production of hydroxyl radicals with time.

The production of H_2_O_2_ in the dark was also investigated. Fig. S9† shows the detection of H_2_O_2_ by Ampliflu Red. A significant fluorescence signal was observed after one hour incubation on the moth-eye imprinted ZnO nanocomposite surfaces. A concentration of H_2_O_2_ in the range of 2–8 μM was detected over an incubation period of 22 h which is deemed to be the main factor causing cytotoxicity to the bacteria cells adhered to the topography. H_2_O_2_ can penetrate the bacteria readily due to a relatively weaker electrostatic repulsion to bacteria than ionic species. As such, H_2_O_2_ has been proposed to be a key factor in the antimicrobial activity producing peroxidation reactions, particularly peroxidation of the bacteria membrane lipids leading to membrane dysfunction.^22,43^

Assessment of the *in vitro* bactericidal activity results obtained on the moth-eye patterned nanocomposites indicates that the addition of ROS active NPs onto the nanocone topography improves remarkably the bactericidal efficiency and allows reducing the NP load onto the material compared to conventional bactericidal nanocomposites. Typically the amount of NPs employed in bactericidal nanocomposites ranges from 2 to 10 wt% while in this case only the active surface is loaded with NPs.

In light of these results, as a hypothetical bactericidal mechanism for the moth-eye patterned nanocomposites, we can postulate that when bacteria attach to these surfaces suffer local mechanical stresses due to membrane stretching and this local stresses increase the susceptibility of the membrane to oxidative damage. Within this hypothetical context, we can assert that the nanopatterned nanocomposites produce a correlated bactericidal action, in which the polymer topography plays an active role by compromising the bacterial membrane aiding to the bactericidal action of NPs.

### 3.4 Cellular toxicity of moth-eye patterned ZnO-nanocomposite

An important requirement of antibacterial materials for the biomedical field is cytocompatibility. For this, antibacterial materials should inhibit the bacterial growth without impacting the viability and growth of eukaryotic cells.^51^ Hence, alongside with the bactericidal activity, the cyto-toxicity toward keratinocytes (HaCat) cells was assayed. Keratinocytes constitute 90% of the epidermis layer and their proliferation and spread as well as their sensitivity to direct contact with surfaces,^52,53^ make these cells good models to test cyto-compatibility.

The attachment and proliferation of HaCaT cell was investigated on the moth-eye patterned ZnO-nanocomposite surfaces. However, because of the UV irradiation requirement for photo-activation, cytotoxicity tests on the TiO_2_-nanocomposites surfaces were not considered relevant.

**Fig. 5:**
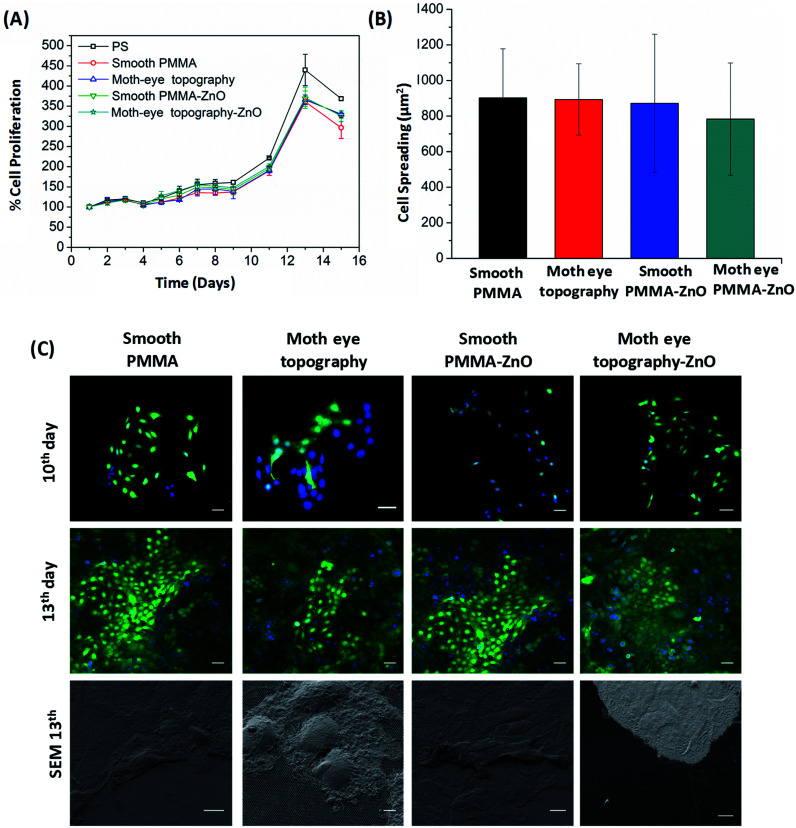
Cytocompatibility properties of moth-eye nanocomposite topography. (A) HaCaT proliferation growth profile on polystyrene, smooth PMMA and moth-eye mimetic nanopatterned nanocomposites. (B) Comparison of the cellular morphology of HaCaT cells seeded on smooth and moth-eye patterned PMMA and on smooth and moth-eye nanopatterned ZnO nanocomposites. The error bars on cell spreading represent standard deviation. (C) Fluorescence Imaging of HaCaT cells after the 10^th^ and 13^th^ day of incubation on smooth and moth-eye patterned PMMA and on smooth and moth-eye patterned PMMA ZnO nanocomposites (Scale bar 50 μm). Below are the corresponding SEM images (Scale bar 10 μm).

During the cyto-toxicity assay, the proliferation of the HaCaT cells was monitored during 15 days on smooth and moth-eye patterned ZnO nanocomposites using as controls smooth and patterned neat PMMA surfaces together with neat polystyrene (PS). PS was included as reference because of its extensive use in *in vitro* cell culture plates. The cell growth profile obtained as depicted in Fig. 5(A), exhibited a typical cell proliferation curve in which cells entered in the growth log phase on the 9^th^ day and reached the maximum growth level at the 13^th^ day. The cells' morphology was obtained from fluorescence images. For this, cells were collected and fluorescently labelled on the 10^th^ and 13^th^ days of growth. Fig. 5(B) shows the analysis of the results. As can it be appreciated, cells on the 10^th^ day appear forming small colonies typical of the HaCaT cell line, and on the 13^th^ day, the cells reached a confluent state in which the substrates appear completely covered by keratinocytes. SEM images of the cells show that effectively, the cells exhibited an extended morphology comparable on all the surfaces. Calculation of the cell spread area revealed no obvious differences in spreading for any of the substrates (Fig. 5(B)). Thus, it appears reasonable to assert that there is no toxic influence on keratinocytes derived from the ZnO NPs at the employed load and neither from the topography and as such, the nanopatterned ZnO-nanocomposites should be suitable to support cell development.

Hence, the moth-eye patterned ZnO-nanocomposite appears to be a promising bactericidal material without cytotoxic effects which can be potentially used in medical devices.

## 4. Conclusion

We have presented the development of a safer-by-design new class of bactericidal materials based on moth-eye mimetic patterned nanocomposites fabricated in continuous processing steps of NP coating and thermal nanoimprinting. The material contains a minimal amount of NPs secured on the active surface. The moth-eye patterned nanocomposites exhibited a remarkable bactericidal activity against Gram positive and Gram negative bacteria. The enhanced bactericidal action derived from a collaborative lethal processes of mechanical stretching induced by moth-eye nanotopography and oxidative stress arising from ROS active ZnO and TiO_2_ NPs. The nanopatterned ZnO–PMMA nanocomposites showed in addition good cytocompatibility, with no significant effect on keratinocytes proliferation or morphology.

Thus, this study presents an industry-relevant, scalable technology that may power a new trend for safer-by-design bactericidal products with reduced risks to the environment and human health and with wide potential fields of application to the biomedical field and also to the consumer care, food packing, furnishing or construction industries.

## Conflicts of interest

The authors declare the following competing financial interests: the authors have applied for a patent on the topic of this paper.

## Supplementary Material

RA-008-C8RA03403F-s001
