# Supplementary material for: Nano-engineering safer-by-design nanoparticle based moth-eye mimetic bactericidal and cytocompatible polymer surfaces
Source: RSC Adv. 2018 Jun 20;8(40):22606–16. doi: 10.1039/c8ra03403f (PMC9081401; doi:10.1039/c8ra03403f)
Supplement: RA-008-C8RA03403F-s001 [file RA-008-C8RA03403F-s001.pdf]

Electronic Supplementary Material (ESI) for RSC Advances.
This journal is © The Royal Society of Chemistry 2018

Electronic Supplementary Information

Nano-Engineering Safer-By-Design Nanoparticle Based Moth-Eye Mimetic Bactericidal and Cytocompatible Polymer Surfaces

*Felipe Viela, Iván Navarro-Baena, Alejandra Jacobo, Jaime J. Hernández, Marta Boyano-Escalera, Manuel R. Osorio, Isabel Rodríguez**

Madrid Institute for Advanced Studies in Nanoscience (IMDEA Nanoscience), C/Faraday 9, Ciudad Universitaria de Cantoblanco, Madrid 28049, Spain
**Correspondence to: i.rodriguez@imdea.org*

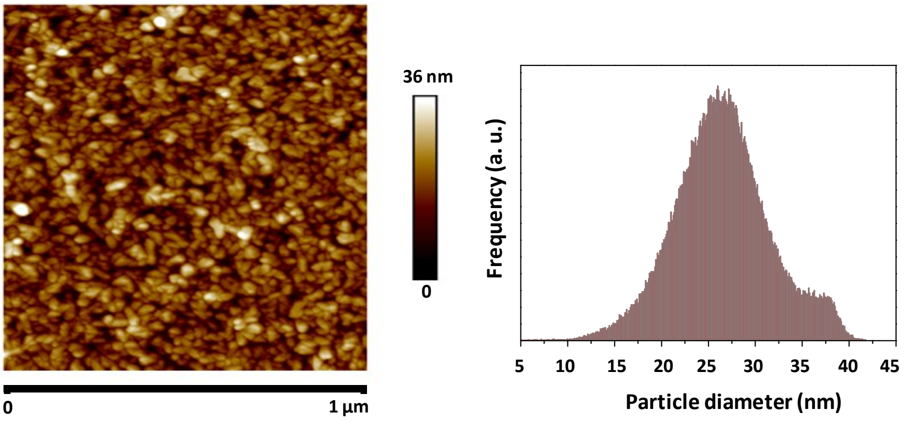

Figure. S1. TiO2 synthesized nanoparticle characterization: AFM image of TiO_2 layered nanoparticles prior imprinting obtained after spin coating a 0.5 wt% TiO_2 dispersion and extracted nanoparticle size distribution from the image (mean nanoparticle size: 26 nm).

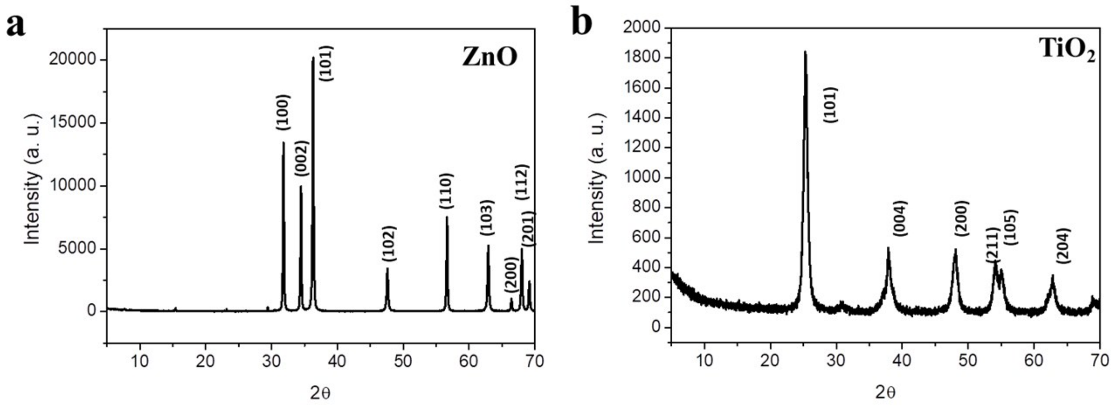

Figure S2. X-ray diffraction patterns of synthesized TiO_2 and ZnO nanoparticles.
The observed diffraction peaks match well with the ZnO wurtzite crystal lattice parameters and with anatase TiO_2 crystal phase structure.

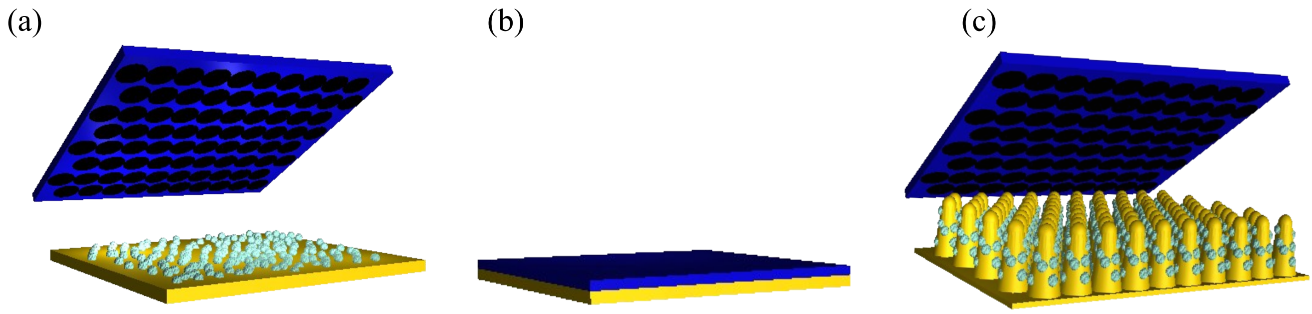

Figure. S3. Schematic illustration of the fabrication process of moth-eye nanocomposite surfaces involving the processes of a) nanoparticle coating, b) thermal nanoimprinting and c) de-molding

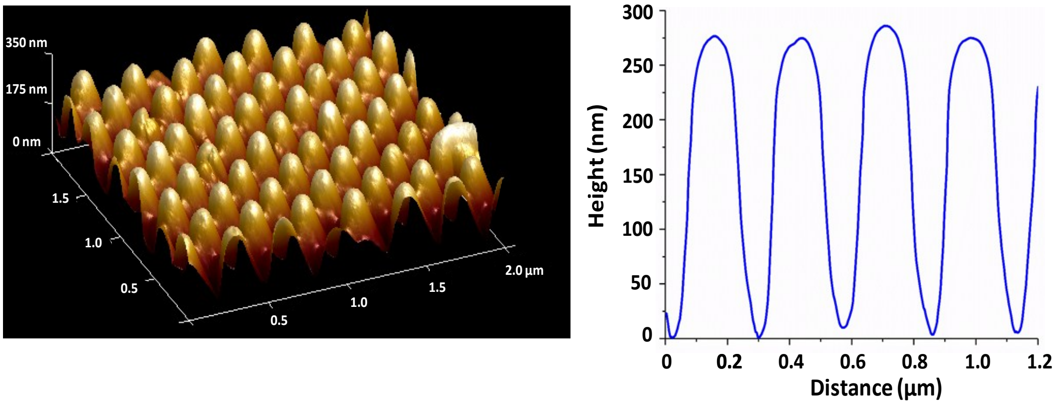

Figure S4. 3D AFM image and cross sectional profile of the imprinted moth-eye mimetic ZnO nanocomposite surfaces.

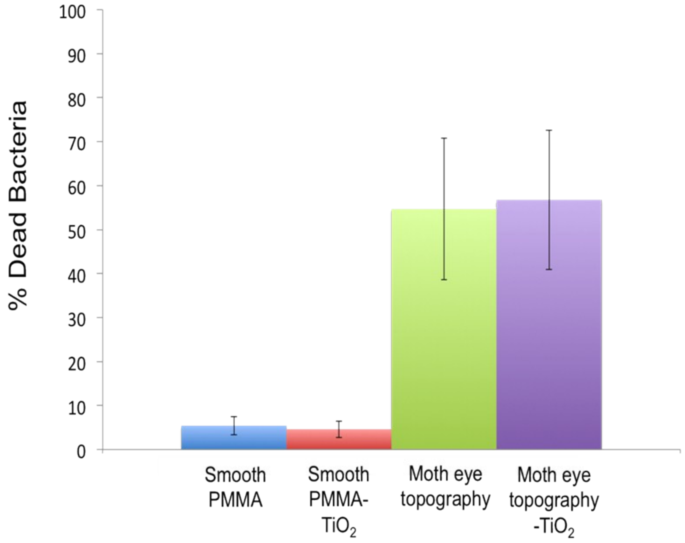

Figure S5. Comparative percentage of dead S. *aureus* cultured on smooth PMMA, smooth PMMA-TiO_2, moth eye topography on neat PMMA and moth eye topography on PMMA-TiO_2 nanocopmposite without UV light exposure. The number of dead bacteria on smooth and nanopatterned titania nanocomposites is comparable to the number of dead bacteria on

smooth and nanopatterned neat PMMA controls, indicating that there is not bactericidal action by the TiO_2 without light activation. The bactericidal action observed in this case corresponds to that effected by the topography.

1. Nanocomposite	Zn^{2+} (µg/l)	Ti^{4+} (µg/l)
*Control(H2O)**	*10.09*	*0.00*
Moth eye ZnO nanocomposite	593.62	0.00
Moth eye TiO_2 nanocomposite	10.09	12.38

2. Nanocomposite	Zn^{2+} (µg/l)	Ti^{4+} (µg/l)
Control(Bacteria culture medium)	2.31	0.00
Moth eye ZnO nanocomposite	41.64	0.00
Moth eye TiO_2 nanocomposite	2.82	0.00

Table S6. Ion release from ZnO and TiO_2 nanocomposites

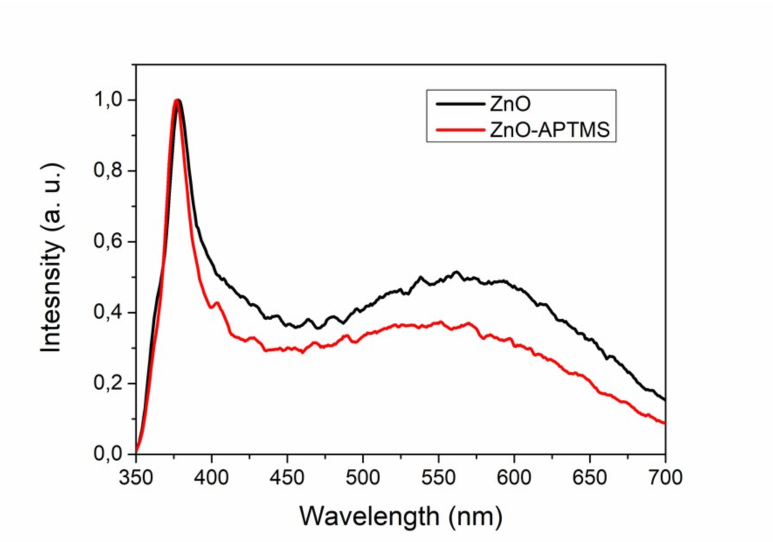

Figure S7 Spectrum of photoluminescence of the ZnO nanoparticles in methanol under 325 nm excitation wavelength at room temperature obtained using a fluorospectrometer (FluoroLog 3, HORIBA). The PL spectrum shows a broad visible luminescence band from 400 to 700 nm originated from the defect states from zinc or oxygen vacancies.[1] The decrease in PL intensity in the visible region upon silanization indicates that the attached silane molecules on the surface of the ZnO nanopartices produce a passivation of the surface defects.

[1] Raji, R., & Gopchandran, K. G. (2017). ZnO nanostructures with tunable visible luminescence: Effects of kinetics of chemical reduction and annealing. *Journal of Science: Advanced Materials and Devices*, *2*(1), 51-58.

Hydroxyl radical detection by the reaction between terephthalic acid and hydroxyl radical with the formation of the fluorescent 2-hydroxyterephthalic acid.

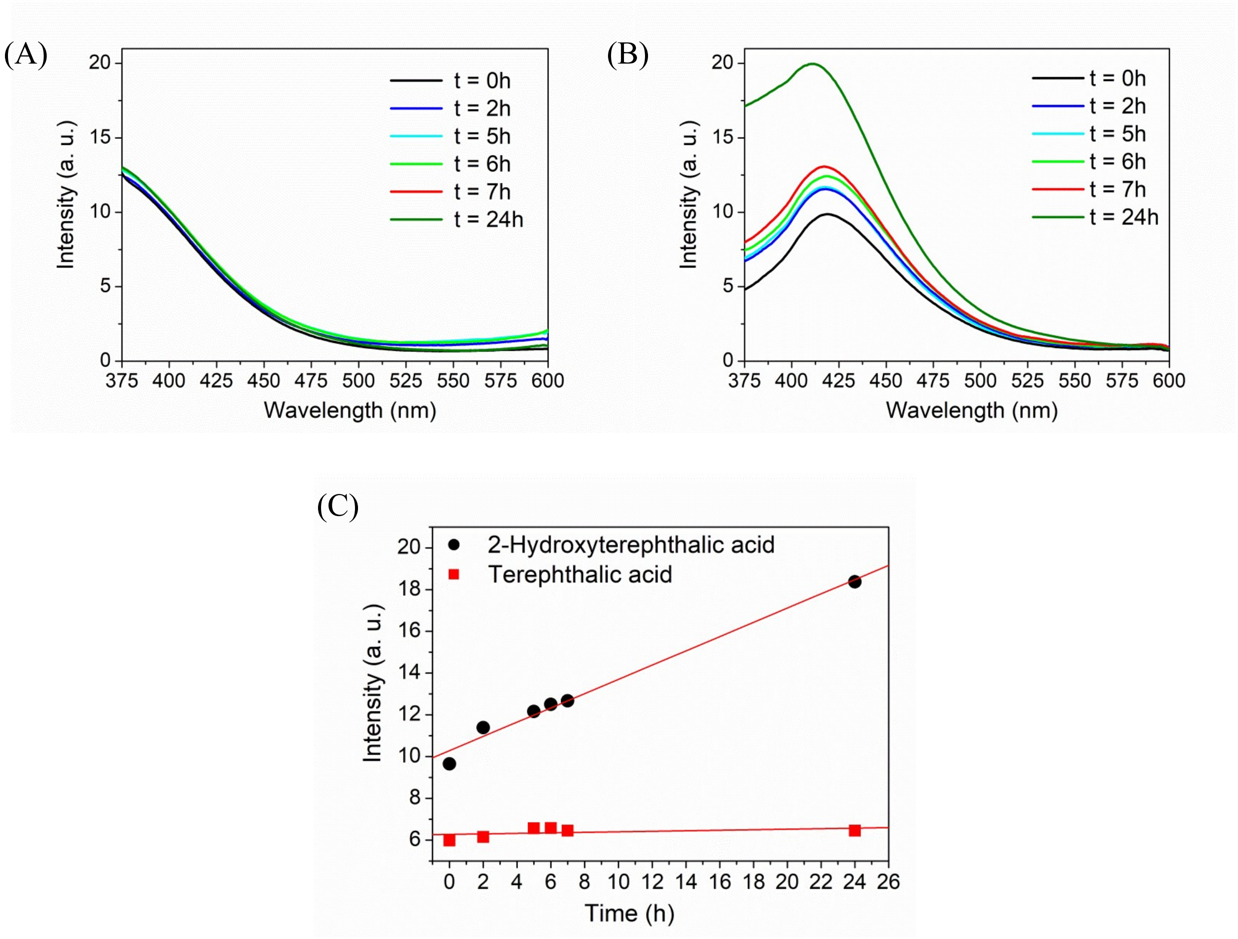

Figure S8. (A) Fluorescence spectrum of hydroxyl terephthalic acid in PB buffer in contact with moth eye ZnO nanocomposite surfaces. The terepthalic acid emission at different times was used as a control. (B) Variation of the 2-hydroxylterepthalic acid fluorescence emission at 425 nm with incubation time on the ZnO nanocomposite surfaces. (C) 2-hydroxyl terepthalic acid formation kinetics

Hydrogen peroxide detection through reaction with Ampliflu red. The assay relies on the reaction of H_2O_2 with the colorless Ampliflu Red to form the fluorescent resorufin in a reaction with a 1:1 stoichiometry catalyzed by horseradish peroxidase (HRP)

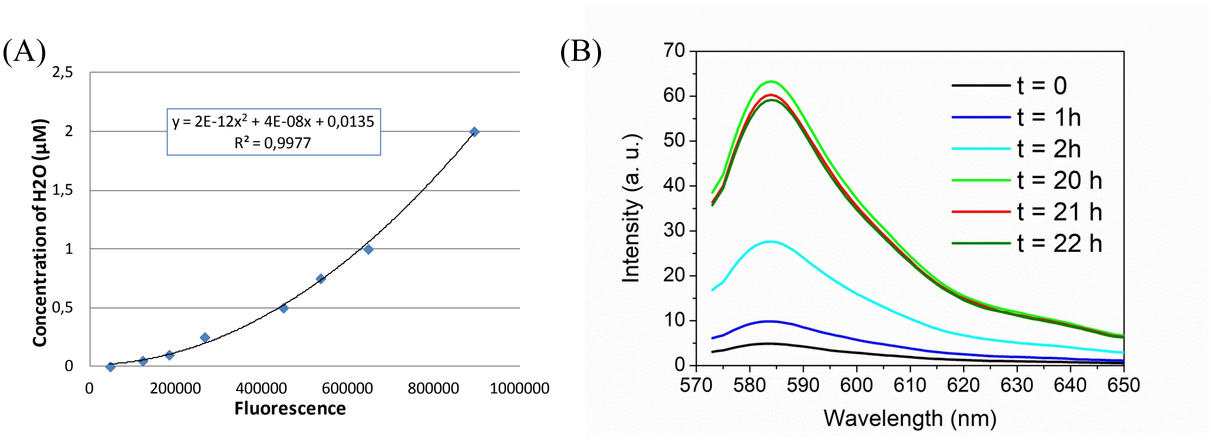

Figure S9. Ampliflu Red assay. (a) H_2O_2 calibration curve. (b) Determination of the H_2O_2 production by the ZnO nanocomposite surfaces in the dark over and an increasing period of time.
